# Distinct Parameters in the EEG of the PLP α-SYN Mouse Model for Multiple System Atrophy Reinforce Face Validity

**DOI:** 10.3389/fnbeh.2016.00252

**Published:** 2017-01-10

**Authors:** Lorenz Härtner, Tobias W. M. Keil, Matthias Kreuzer, Eva Maria Fritz, Gregor K. Wenning, Nadia Stefanova, Thomas Fenzl

**Affiliations:** ^1^Department of Pharmacology and Toxicology, Institute for Pharmacy, Leopold-Franzens University of InnsbruckInnsbruck, Austria; ^2^Neuroanesthesia Laboratory, Atlanta Veterans Affairs Medical Center/Emory University and Department of Anesthesiology, Emory UniversityAtlanta, Georgia; ^3^Department of Neurology, Medical University InnsbruckInnsbruck, Austria

**Keywords:** multiple system atrophy, sleep, EEG, mouse model, predictive validity

## Abstract

Multiple system atrophy (MSA) is a neurodegenerative movement disorder characterized by parkinsonian symptoms and cerebellar symptoms. Sleep disturbances also play a crucial role in MSA. One of the most convincing animal models in MSA research is the PLP α-SYN model, but to date no studies on sleep disturbances in this mouse model, frequently found in MSA patients are available. We identified spectral shifts within the EEG of the model, strikingly resembling results of clinical studies. We also characterized muscle activity during REM sleep, which is one of the key symptoms in REM sleep behavioral disorder. Spectral shifts and REM sleep-linked muscle activity were age dependent, supporting Face Validity of the PLP α-SYN model. We also strongly suggest our findings to be critically evaluated for Predictive Validity in future studies. Currently, research on MSA lacks potential compounds attenuating or curing MSA. Future drugs must prove its potential in animal models, for this our study provides potential biomarkers.

## Introduction

Multiple system atrophy (MSA) is a sporadic and rapidly progressive neurodegenerative movement disorder characterized by parkinsonian (MSA-P) symptoms and cerebellar symptoms (MSA-C) (Gilman et al., 2008; Fanciulli and Wenning, 2015). Non-motor features including autonomic and sleep disturbances also play a crucial role in MSA that significantly reduce patients' quality of life. Epidemiologic studies report a disease probability of 0.6–3/100.000 patients per year (Bower et al., 1997). The mean disease onset is around 60 years and both sexes are affected equally with less than 9 years survival time after onset (Schrag et al., 2008; Kuzdas et al., 2013). Ethnic differences exist in the expression of the motor sub-forms. In Europe, MSA-P prevails in 58% of cases reported (Geser et al., 2006) and in North America 60% of MSA patients develop MSA-P symptoms (May et al., 2007). In contrast to that, more than 83% of reported MSA cases are diagnosed as MSA-C in Japan (Yabe et al., 2006). Currently, symptomatic treatment is restricted to autonomic and parkinsonian features (Papatsoris et al., 2008). On a neuropathological level, neurodegeneration affects multiple brain areas including the basal ganglia, cerebellum, pontine, and inferior olivary nuclei, pyramidal tract, intermediolateral cell column, and Onuf's nucleus. The neuronal degeneration is often accompanied by gliosis (Wenning et al., 2004). MSA is also classified among the α-synucleinopathies because of α-synuclein (αSYN)-immunoreactive inclusion bodies in oligodendrocytes, so-called glial cytoplasmic inclusions (GCI) (Wenning et al., 2008).

Intriguingly, *sleep disturbances*, as described below are common in MSA patients affecting sleep regulation at multiple levels, reflecting the diffuse underlying neuropathological processes involved in MSA (Ghorayeb et al., 2002). Disturbances of sleep include insomnia, excessive daytime sleepiness (EDS), restless legs syndrome, rapid eye movement sleep (REMS), behavior disorder (RBD), sleep related breathing disorders and nocturnal inspiratory stridor. On polysomnographic examination, MSA patients show disturbed sleep architecture including sleep fragmentation and REMS behavior disorder, including REMS without atonia (REM-A) (Wetter et al., 2000; Vetrugno et al., 2004).

*RBD* is a parasomnia characterized by a loss of normal skeletal muscle atonia during REMS (REM-A, based on the nomenclature by Olson and Schenck (Olson et al., 2000; Schenck et al., 2002; Schenck and Mahowald, 2002). REM-A is a polysomnographic finding and if combined with nightmares (of mostly violent content) and dream enactment behavior, REM-A is referred to as RBD. Among all etiologies RBD is most common in MSA. In fact, the frequency of RBD is 0.05% among the general population, however, virtually every MSA patient suffers from RBD (Plazzi et al., 1997; Tachibana et al., 1997; Vetrugno et al., 2004; Scaglione et al., 2005), first described by Tison et al. (1995). RBD is frequently the first non-motor manifestation of neurodegenerative α-synucleinopathies (Schenck and Mahowald, 1996; Boeve et al., 1998; Iranzo et al., 2006; Claassen et al., 2010). The first study to document the relationship of RBD and neurodegenerative diseases reported that in almost 40% of patients with isolated, idiopathic RBD, it was accompanied by α-synucleinopathy within a 10-year period (Schenck and Mahowald, 1996). This finding was confirmed by several longitudinal investigations in other RBD cohorts (Iranzo et al., 2006; Postuma et al., 2009), linking RBD as an early risk factor to the development of α-synucleinopathies (Arnulf, 2012). Interestingly, two recent studies demonstrated that in contrast to the waking state, movements during RBD were comparably fast without tremor and bradykinesia, providing an insight into motor control during REMS (De Cock et al., 2007, 2011). We know of only one case report in the literature investigating the longitudinal course of MSA-RBD (Tachibana and Oka, 2004). The study demonstrated that RBD related behavior decreased over time and disease duration, whereas the percentage of REM-A increased.

*Restless leg syndrome* (RLS) is a common neurological disorder that is accompanied by *periodic limb movements in sleep* (PLMS), which can be observed in up to 88% of MSA patients (Plazzi et al., 1997; Wetter et al., 2000; Vetrugno et al., 2004; Ghorayeb et al., 2005). RLS is characterized by an unpleasant restlessness, mostly in the legs associated with an urge to move (Trenkwalder, 1998), worst at rest, relieved by attempts to move, and most disturbing in the evenings (Allen et al., 2003).

*Sleep fragmentation* is a further very common sleep-related disturbance among MSA patients (Ghorayeb et al., 2002). Sleep fragmentation is manifested by in an increased number of short arousals or awakenings and a deficiency in consolidated sleep. It occurs during periods of non-rapid eye movement sleep (NREMS) and especially in Parkinson's Disease also in REMS (Högl et al., 1998).

*The animal model for MSA in the present study*: Several approaches for MSA animal models exist in mouse and rat, including lesions of nigrostriatal brain regions, injections of toxins, transgenic models or combinations of these individual techniques (Stefanova et al., 2005a). One of the most convincing animal models in present research is the so-called PLP α-SYN model, a PLP (proteo-lipid-protein promotor) α-synuclein mouse strain (Kahle et al., 2002). Immunohistochemical and microscopic studies revealed concordant pathology between human and murine probes at a cellular level. Hyper-phosphorylated α-SYN was found in MSA patients and transgenic mice, the inclusions were similarly half-moon- or triangulated-shaped and present in the cytosol around the nucleus. The α-SYN was detergent insoluble, which is a diagnostic criterion for α-synucleiopathies (Kahle et al., 2002). Behavioral phenotyping performed with this animal model revealed coherence with key symptoms of MSA in humans: Urodynamic analysis showed a less efficient and unstable bladder activity with an increased voiding contraction amplitude, a higher frequency of non-voiding contractions and an increased post-void residual volume (Boudes et al., 2013). Other non-motor symptoms were a decreased heart rate variability (Kuzdas et al., 2013) and respiratory dysfunction (Flabeau et al., 2014). The PLP α-SYN model also showed shorter stride length, deficits on pole rod and beam walking tests as well as changes in grip strength and gait variability (Stefanova et al., 2005a,b).

However, the present animal model may not reflect the entire pathology of human MSA. Especially the temporal distribution of neuronal degeneration and neuronal loss in relevant brain regions (Stefanova and Wenning, 2015) may be different. This may also be true at the behavioral level. So far no studies were performed to characterize sleep disturbances in the PLP α-SYN model, common in MSA patients. Potential candidates of sleep disturbances in the animal model could be RBD with REM-A, RLS or sleep fragmentation. On one hand, such findings would strengthen Face Validity for the PLP α-SYN model. On the other hand, if sleep disturbances would develop longitudinally along the life span of the animal model, such behavioral manifestations could serve to establish Predictive Validity for drug screenings and evaluation of therapeutic approaches.

## Materials and methods

### Animal numbers

Male transgenic homozygous mice overexpressing human a-synuclein under the proteolipid protein (PLP) promotor (the generation and characterization of the mice has been previously described in detail (Kahle et al., 2002) and further referred to as PLP-aSYN or MSA mice) and age-, sex-, and background-matched wildtype C57BL/6 (control) mice were used in this study. The control group consisted of 8 mice at an age of 11–18 weeks (mean BL6_young_ = 14 weeks/±SEM = 1.03) and 8 mice at an age of 38–49 weeks 8 (mean BL6_adult_ = 40.83 /±SEM = 1.70). The MSA group consisted of 8 mice at an age of 14–18 weeks (mean MSA_young_ = 16/±SEM = 0.46) and 10 mice at an age of 38–49 weeks (mean MSA_adult_ = 45.40 /±SEM = 0.73). No significant age differences were detected in MSA_young_ vs. BL6_young_animals (*t*-test, *P* = 0.085). We had to exclude two BL6_adult_ mice as well as one BL6_young_ animal from our analysis due to low EMG signal quality during signal processing for REM-A analysis. All transgenic mice (number of backcrossing over 50) were homozygous. Before transferred to the laboratory, the animals were genotyped for α-syn by tail clip PCR using following primers with a product size of 450 bp: fwd: 5′-ATG GATGTATTCATGAAAGG-3′; rev: 5′-TTA GGCTTCAGGTTCGTAG-3′ (Kahle et al., 2002).

### Housing conditions

After transferring the mice from the animal facility to the laboratory, we placed each animal in an individual home cage (custom made) with access to water and food *ad libitum*. Throughout the study two home cages were always kept in a custom made sound attenuated chamber (olfactory and visual contact). We allowed the animals to adapt to the day/night cycle of the experiments (lights ON: 10 a.m./lights OFF: 10 p.m.; temperature: 23°C ± 1°C) for 7 days before implantation of the recording electrodes, followed by a 14 day recovery period. During recovery, we permanently kept two animals in individual home cages in an electrically shielded (bench top Faraday cage, Peabody, USA) and sound attenuated recording chamber (custom made). We connected each animal to a pre-amplifier (amplification: 1x, npi electronics, Tamm, Germany) to allow for adaption 3 days before we started the chronic recordings that typically lasted 3 consecutive days (Fenzl et al., 2007). After termination of the experiment we sacrificed the animals by perfusion with 4% PFA and stored the brains at −80°C for further analyses.

### Surgical procedure

We anesthetized the animals with 1.9–2.2% isoflurane at a flow rate of 200 ml/min (U-410, agnthos, Lidingö, Sweden). A feedback-controlled heating pad kept the body temperature at 37.8°C (cma450, Harvard Apparatus, USA). At a sufficient level of anesthesia we fixated the animals in a stereotaxic frame, shaved the head, opened the scalp medially, and removed the periosteum. We used a dental precision driller (Typ 4911, KaVo, Germany) to drill six holes into the skull and drove two Jeweler's screws (diameter: 150 μm) in the median two holes to fixate the implantations. The EEG electrodes were placed in the left and right part of the frontal bone (from Bregma/rostral: +1 mm, medio-lateral: ±1 mm) and the grounding and reference electrode in the parietal bone (from Bregma/caudal: −2.5 mm, medio-lateral: ±1.8 mm). Two EMG electrodes were lowered bilaterally into the neck muscle, directly caudal to the occipital bone. All recording electrodes, consisting of gold wire (diameter: 150 μm, Häfner, München, Germany) with ball-shaped endings were soldered to a PCP socket board (Type 861-87-008-10-001101, preci-dip, Switzerland), which was later connected to the pre-amplifier and the recording cable. For a detailed description of the surgical procedure and electrode design, please refer to Fulda et al. (2011) and Polta et al. (2013). During and after surgery, animals received analgesic treatment (Meloxicam, 0.5 mg/ml suspension, s.c. injection and 5 × 10^−4^ mg/ml for 7 days in drinking water).

### Data recording and processing

We attached the pre-amplifiers to the socket boards, mounted on each animal's head and to a commutator (SL-10, Dragonfly, Ridgeley, USA), which was mounted on a weight-neutral swivel system (custom made, Streicher M., Innsbruck, Austria) to allow the animal free movement in all three dimensions during the chronic recording sessions (video monitored). We recorded for 23 h in one session and used hour 24 for animal care and housing maintenance before starting the next recording session. Each recorded channel was individually amplified (amplifier type: DPA-2FL, npi electronics, Tamm, Germany), band-pass filtered (amplifier-hardware filter before digitization: 0.1–100 Hz for EEG and 50–90 Hz for EMG/gain: 1000x), and sampled with 250 Hz (POWER1301-1, CED, Cambridge, Great Britain). We used Spike2 Software (Version 7, CED, Cambridge, Great Britain) to record and store the digitized data. We applied a semi-automated sleep scoring software developed by the authors (Kreuzer et al., 2015) to assign the vigilance states WAKE, NREMS, and REMS to non-overlapping 4 s EEG episodes (1 epoch = 4 s). This analysis software is based on algorithms published for sleep analysis in rats (Louis et al., 2004), adapted for mice (Fenzl et al., 2007). A scorer blind to the data sets manually reviewed all semi-automated sleep scorings at epoch-level. We only considered epochs of a defined vigilance state that lasted longer than 3 epochs (12 s) for a change in the behavioral status. Consequently, we attributed vigilance changes that lasted shorter than three epochs only to micro arousals within the EEG.

### Data analysis

We analyzed the EEG data of the single animals in multiple ways. For an overall impression of the animal's sleep behavior, we present the proportions of the different vigilance states WAKE, NREMS, and REMS for the experimental groups (BL \6: young and adult; MSA: young and adult). Then we derived the mean duration each animal spent in any given vigilance state (bout length) to gain quantitative and qualitative insight in the fragmentation of vigilance states in the experimental groups. Additionally we analyzed the number of transitions between the vigilance states in each experimental group with five possible transitions between WAKE, NREMS, and REMS (the transition from WAKE to REMS is behaviorally not present). We also analyzed spectral parameters of the EEG [e.g., power spectral density (PSD)] to detect qualitative differences in the EEG properties of the experimental groups. For that we down-sampled the EEG recorded at 250–125 Hz and estimated the power spectral density for non-overlapping 4 s episodes with the MATLAB *pwelch* function. Additionally the “Slow wave activity” (SWA-PSD: 0.5–5 Hz) during NREMS was evaluated. In a last step, we searched manually at epoch-level for the occurrence of REM-A episodes (REMS episodes where the animal clearly expressed EMG activity).

### REM-A analysis

We defined REMS without atonia (REM-A) as a single epoch or multiple epochs of REMS with simultaneous neck muscle activity, recorded from the EMG electrodes. For that, we re-analyzed the corresponding EMG activity for each single REMS epoch identified by the semi-automatic sleep scoring and/or manual re-scoring. The re-analysis was necessary to detect REM-A epochs/episodes that were scored as WAKE during the semi-automatic process of sleep scoring due to EMG-activity which is defined as WAKE *per se* in our scoring routines (Fenzl et al., 2007, 2011; Polta et al., 2012; Kreuzer et al., 2015). We only assigned muscle activity to REM-A if the EEG could be unmistakably discriminated from an WAKE EEG (different scorers blind to each other and to the data sets).

### Statistical analysis

**Figure 1:**
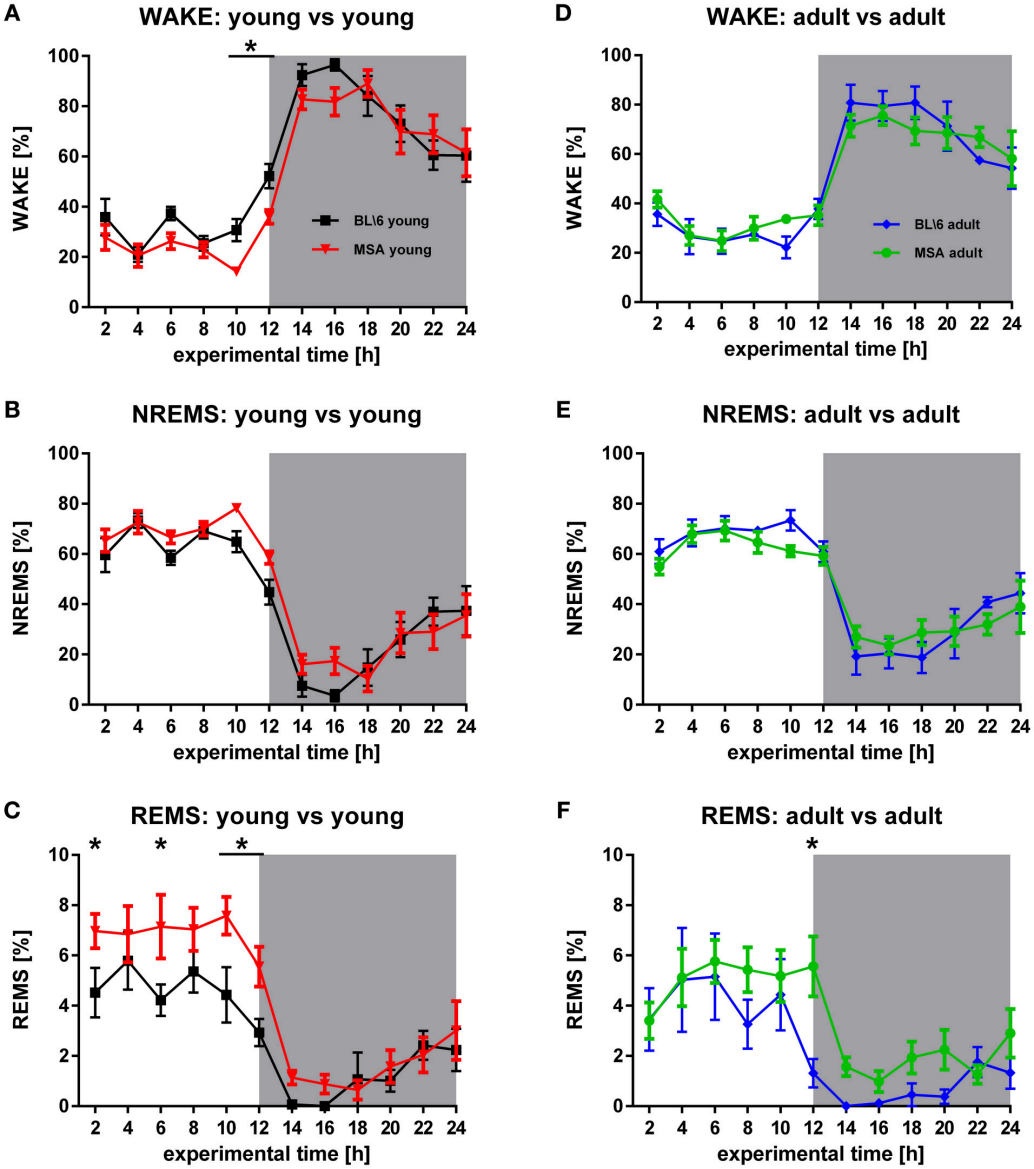
**Age-matched sleep behavior in MSA mice vs. control animals. (A,B,D,E)** WAKE and NREMS is generally very similar in young MSA mice and young control animals (*F*_Wake_ = 5.997, *p* = 0.015; *F*_NREMS_ = 5.997, *p* = 0.015) and adult (*F*_Wake_ = 0.0000768, *p* = 0.993; *F*_NREMS_ = 0.321, *p* = 0.572). **(C,F)** Significant differences between MSA mice and control animals in REMS, detected around 10 (*F* = 17.336, *p* < 0.001) clearly decrease in adult mice (*F* = 10.397, *p* = 0.002). For all graphs shown: X-axis represents the experimental time of one 24 h-recording session, Y-axis represents the amount of the behavioral state WAKE, NREMS, and REMS. White background: inactive period/lights on (hour 0 to hour 12), gray background: active period/lights off (hour 12 to hour 24). All data are 2 h means ± SEM, Two way ANOVA, followed by Bonferroni corrections (*p* ≤ 0.05). Number of animals: n_MSA_young = 8, n_MSA_adult = 10, n_Control_young = 8, n_Control_adult = 6.

For the comparison of the sleep behavior between the MSA and the control animals we used *two-way repeated ANOVA tests* (SigmaStat 3.5, Systat, Erkrath, Germany). The factors were “strain” and “time.” We further used *two-way repeated ANOVA tests* with factors “age” and “time” to evaluate the impact of age on the sleep behavior within the strain. We further used this statistical test to check for differences in the SWA spectral power with factors “age” and “time.” For the evaluation of possible differences in the power spectral density between the groups at different vigilance levels, we used a *two-way ANOVA with Bonferroni correction*. Our *post-hoc* test of choice was the *Bonferroni correction* to correct for multiple comparisons (*p* < 0.05). We applied the concept of cumulative probability to the bout length analysis and used the two *sample Kolmogorov-Smirnov test* to check for differences between the distributions of bout lengths (MATLAB R2015a, MathWorks, Natick, Ma, USA). We further used the two *sample Kolmogorov-Smirnov test with Bonferroni correction* to evaluate possible difference in state transition probabilities. In order to evaluate differences in REM-A among the groups and to evaluate the separating performance of REMS amount (Figures 1C,F) using Youden's index, we performed an *area under the receiver operating curve* (AUC) analysis including 95% confidence intervals [95% ci; AUC values adjusted to the interval (0.5–1)]. We performed these tests using the MATLAB-based MES toolbox (Hentschke and Stüttgen, 2011) with the following considerations: AUC > 0.64 a medium effect and AUC > 0.71 a strong effect (Rice and Harris, 2005).

All experiments were performed in accordance with the international and national guidelines and were approved by the “Bundesministerium für Wissenschaft, Forschung und Wirtschaft, Austria (BMWFW-66-008/0011-WF/V/3b/2014) and the animal welfare committee of the LFU (Tierschutzgremium LFU).

## Results

### Sleep architecture

#### Inter-group analysis

The circadian distribution of sleep and WAKE in MSA and control animals represented a typical distribution of the vigilance states WAKE, NREMS, and REMS, as expected for nocturnal animals (Figure 1). During the inactive period (lights on), both groups showed low levels of WAKE, that strongly increased in the active period (lights off). Consequently, we observed high (low) proportions of NREMS in the inactive (active) period. We observed this distribution of vigilance states in all four groups (Figures 1A–E). The distribution of the WAKE proportions over 24 h was significantly different in the young animals (ANOVA: *p* = 0.015) with a significantly higher proportion of WAKE directly before turning the lights off in the BL\6 mice (Figure 1A). We found no difference between the old BL\6 and old MSA mice (Figure 1D). We further observed differences in the NREMS proportions in the young mice (ANOVA: *p* = 0.015; Figure 1B), but not in the aged animals (Figure 1E). We found a significantly different distribution of REMS in the young animals (ANOVA: *p* < 0.001) with significantly higher amounts of REMS in young MSA animals during the inactive period (Figure 1C). ROC analysis of REMS amounts, averaged over the inactive period revealed an AUC of 0.80 (95% ci: 0.51–1) with a sensitivity = 0.71 and a specifity = 0.86 at the maximum Youden's index. We also found a difference in REMS distribution in the aged animals (ANOVA: *p* = 0.002), but the *post-hoc* analysis revealed an increased REMS only during the 12^th^ h.

#### Intra-group analysis

**Figure 2:**
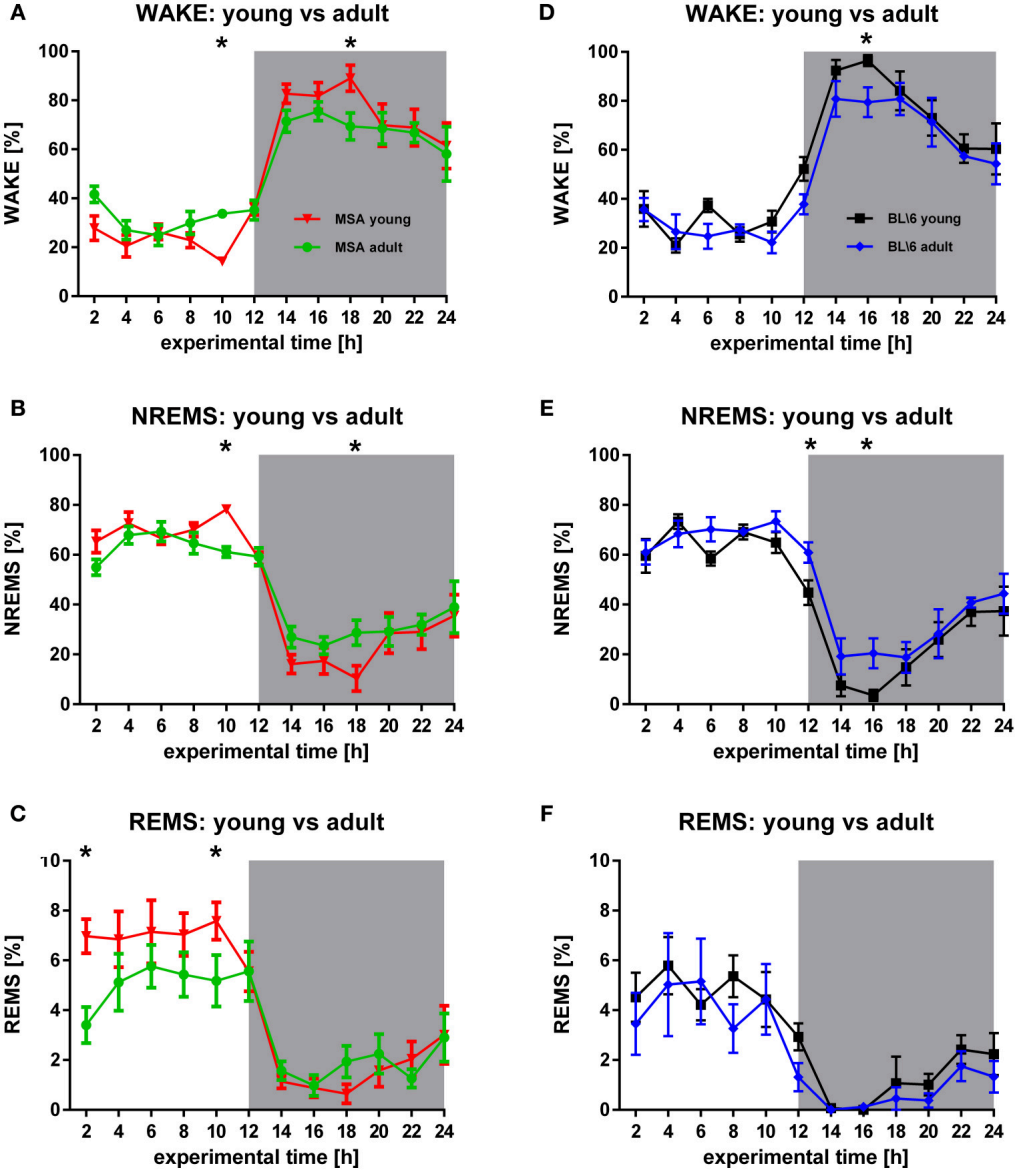
**Longitudinal sleep behavior in MSA mice and BL6 control animals. (A,B)** Young MSA mice have significant differences in WAKE (*F* = 0.0118, *p* = 0.914) and NREMS (*F* = 0.272, *p* = 0.602) in the inactive period (hour 10) and the active period (hour 18), when compared with adult MSA animals. **(C)** Although, only significant at hour 2 and hour 10, the overall REMS behavior implicates behavioral differences in REMS in adult MSA animals vs. young MSA mice during the inactive period (*F* = 6.161, *p* = 0.014). **(D,E)** Similar to MSA mice, control animals have significant differences in WAKE (*F* = 5.955, *p* = 0.016) (hour 16) and NREMS (*F* = 7.947, *p* = 0.006) (hour 12 and hour 16). **(F)** No differences could be detected for control mice during REMS (*F* = 7.947, *p* = 0.006). For all graphs shown: X-axis represents the experimental time of one 24 h-recording session, Y-axis represents the amount of the behavioral state WAKE, NREMS, and REMS. White background: inactive period/lights on (hour 0 to hour 12), gray background: active period/lights off (hour 12 to hour 24). All data are 2 h means ± SEM, Two way ANOVA, followed by Bonferroni corrections (*p* ≤ 0.05). Number of animals: n_*MSA*_young = 8, n_*MSA*_adult = 10, n_Control_young = 8, n_Control_adult = 6.

The circadian distribution of WAKE and NREMS changed in both animal groups during aging to some degree (Figures 2A,B,D,E). These differences were present in the control group only during the active period (Figures 2D,E), while MSA animals could be differentiated also during the inactive period (Figures 2A,B). Seniors of both animal groups spent more time in NREMS during the active period. Clearly, old MSA mice spent less time in REMS during the inactive period, when compared with young animals of the same group (Figure 2C). We found no differences between young and old control animals for REMS (Figure 2F).

#### Inter/intra-group analysis/bout length

**Figure 3:**
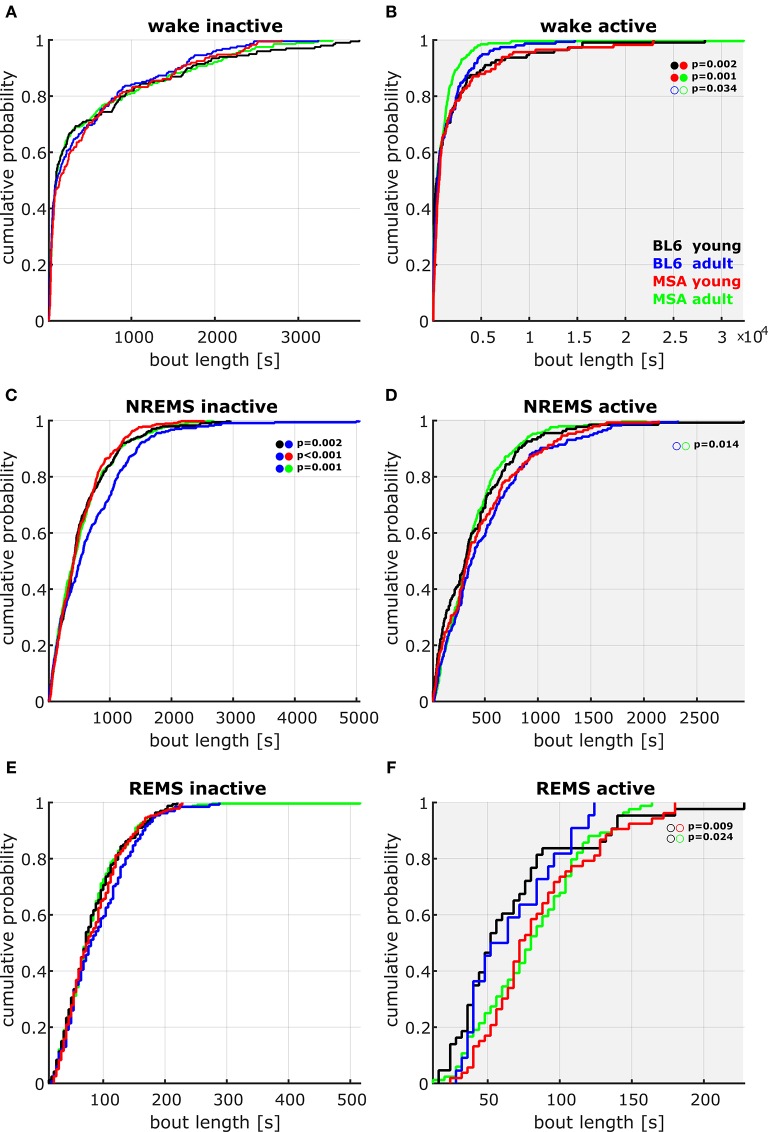
**Cumulative probability plots of the pooled data containing the bout lengths in the different vigilance states during active and inactive periods. (A,B)** Only in the active period (lights off, **B**), young MSA mice showed a different distribution of WAKE bout length in comparison to adult MSA and young BL\6 animals. Here the young MSA animals had higher probability of WAKE episodes of long duration. Adult MSA mice tend to have shorter WAKE episodes in the active period. **(C,D)** During the inactive period (lights on, **C**), the distribution of NREMS bout length in adult control animals was significantly different to all other groups, with an increased probability of longer NREMS episodes (two sample Kolmogorov Smirnov test with Bonferroni correction (full circles); empty circles: *p* < 0.05). No significant differences could be detected during REMS, although during the active period data were not as consistent as during the inactive period.

We found some differences in the distribution of the bout lengths, i.e., how long the animals remained in a vigilance state once they entered it. During the active period, the young MSA animals had a different distribution of WAKE bouts than young BL\6 or aged MSA mice (Kolmogorov-Smirnov: *p* = 0.002 vs. young BL\6; *p* = 0.001 vs. aged MSA). Aged BL\6 animals expressed a significantly different distribution of NREMS bouts during the inactive period. (Kolmogorov-Smirnov: *p* = 0.002 vs. young BL\6; *p* < 0.001 vs. young MSA; *p* = 0.001 vs. aged MSA). Figure 3 displays the cumulative distribution plots for all vigilance states during the active and inactive period.

#### Inter/intra-group analysis/transition frequencies between vigilance states

**Figure 4:**
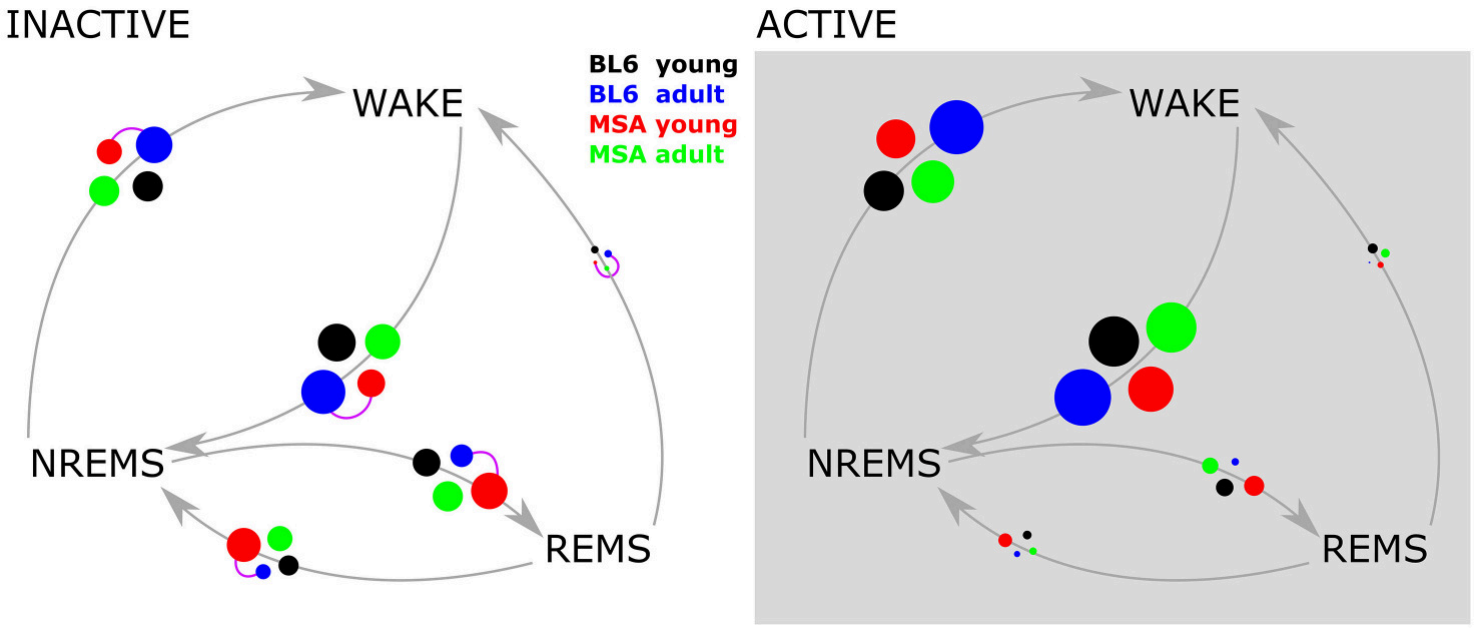
**Vigilance state transition diagrams in the active and inactive period**. The four experimental groups are represented by the colored circles. The circle size corresponds to the probability of the state transition occurring (gray arrows), derived from pooled data. Purple connector lines between the circles indicate significant differences in the distribution of the transition (two sample Kolmogorov Smirnov test with Bonferroni correction).

We only observed significant differences in the transition frequency between vigilance states in the inactive period and only between aged BL\6 and young MSA animals. During the inactive period the animals showed an increased proportion of transitions between NREMS and REMS as compared to the active period, where the animals predominantly transitioned between WAKE and REMS. The corresponding bubble plots in Figure 4 display the detailed transitioning behavior of all four animal groups.

#### REMS latency

While both control groups expressed coherent REMS not before around 2000 epochs into the active period (ca. 130 min after the transition from light to dark), both MSA groups showed REMS directly after the transition from light to dark. Additionally, we only found this phenomenon during the active period, while during the inactive period REMS latency was not different among all four animal groups (please refer to Supplementary Figures 1, 2 for details).

### Spectral analysis

**Figure 5:**
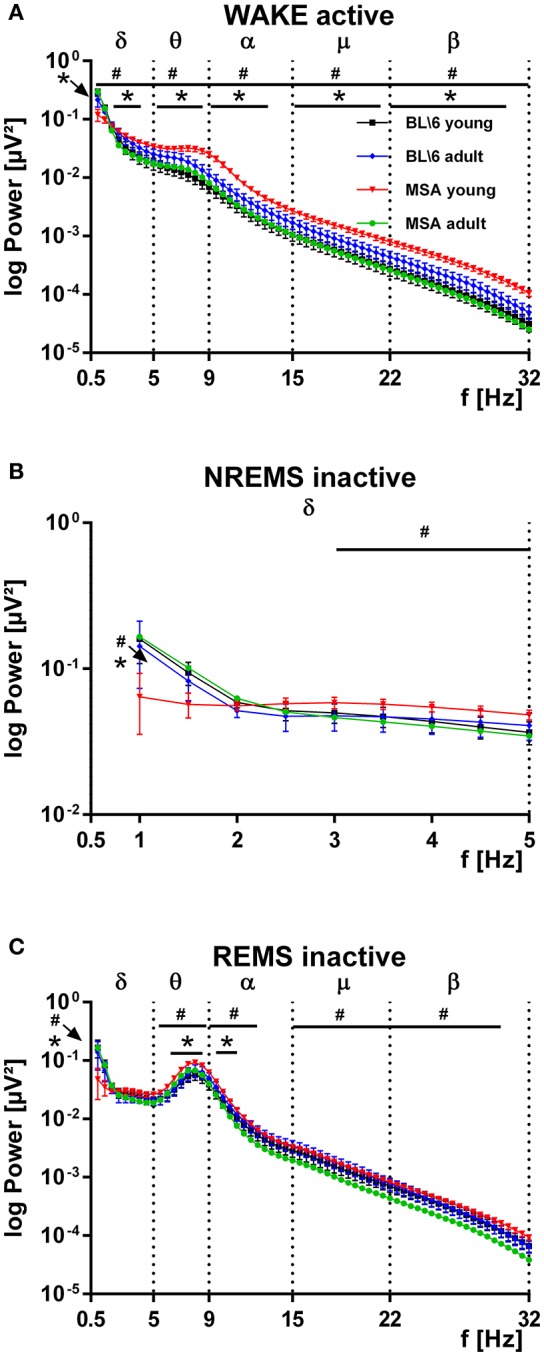
**Distribution of the power of distinct frequency bands in the EEG of MSA animals and control animals. (A)** The power spectrum of the recorded EEGs, subdivided into distinct frequency bands (δ, θ, α, μ, β) is significantly increased in young MSA animals, compared to all three other animal groups. Clearly in adult MSA mice, the power spectrum approached values of the young and adult control groups. The young and adult control groups can hardly be distinguished. ANOVA: MSA_young_ vs. control_young_: *F*_δ_ = 6.032, *p* = 0.015; *F*_θ_ = 186,668, *p* < 0.001; *F*_α_ = 232,338, *p* < 0.001; *F*_μ_ = 252,450, *p* < 0.001; *F*_β_ = 21.779, *p* < 0.001. MSA_young_ vs. MSA_adult_: *F*_δ_ = 39.451, *p* < 0.001; *F*_θ_ = 252.690, *p* < 0.001; *F*_α_ = 392.296, *p* < 0.001; *F*_μ_ = 545.561, *p* < 0.001; *F*_β_ = 720.189, *p* < 0.001. **(B)** During slow wave activity (SWA, δ-band) from 0.5 to around 2 Hz the power of the EEG in young MSA mice is below values of all three other animal groups, while from 3 to 5 Hz the EEG-power of young MSA animals is constantly above values of all other groups. ANOVA: MSA_young_ vs. control_young_: *F*_δ_ = 1.023, *p* = 0.314; *F*_θ_ = 29,887, *p* < 0.001; *F*_α_ = 37,524, *p* < 0.001; *F*_μ_ = 39,634, *p* < 0.001; *F*_β_ = 69.349, *p* < 0.001. MSA_young_ vs. MSA_adult_: *F*_δ_ = 5.637, *p* = 0.019; *F*_θ_ = 99.557, *p* < 0.001; *F*_α_ = 157.661, *p* < 0.001; *F*_μ_ = 280.615, *p* < 0.001; *F*_β_ = 440.146 *p* < 0.001. **(C)** The EEG-power of the relevant frequency band for REMS is constantly above values from all other groups. #: MSA young vs. MSA adult; ^*^: MSA young vs. control mice young. All data are 0.5 Hz means ± SEM, Two way ANOVA, followed by Bonferroni corrections (*p* ≤ 0.05). Number of animals: n_MSA_young = 8, n_MSA_adult = 10, n_Control_young = 8, n_Control_adult = 6. ANOVA: MSA _young_ vs. control _young_: *F*_δ_ = 1.208, *p* = 0.274; *F*_θ_ = 51,050, *p* < 0.001; *F*_α_ = 17,819, *p* < 0.001; *F*_μ_ = 6961, *p* = 0.009; *F*_β_ = 13.377, *p* < 0.001. MSA_young_ vs. MSA_adult_: *F*_δ_ = 0.456, *p* = 0.501; *F*_θ_ = 96.382, *p* < 0.001; *F*_α_ = 201.180, *p* < 0.001; *F*_μ_ = 454.495, *p* < 0.001; *F*_β_ = 438.324, *p* < 0.001.

We analyzed the spectral power from the bandpass-filtered EEG signals (δ, θ, α, μ, β bands) and found most significant differences for WAKE during the active period (Figure 5A), for the slow wave activity (SWA: 0.5–5 Hz) in NREMS in the inactive period (Figure 5B) and for REMS during the inactive period (Figure 5C). Young MSA mice had significantly increased levels of spectral power almost across the whole frequency band. This was found for young MSA against old MSA mice (hash marks) and for young MSA mice against young control mice (asterisks). We found no such differences for young vs. old control animals. Importantly, no differences could be detected between old MSA animals and both control groups (Figure 5A). The increase of power in the second half of the θ-band and the first half of the α-band in young MSA mice was attenuated and shifted to the left covering only the θ-band in old MSA mice and both control groups. During SWA, young MSA mice had decreased power between 1 and 2 Hz, followed by increased power between 3 and 5 Hz, when compared to all other animal groups. No differences in spectral parameters were detected in old MSA, young and old control animals (Figure 5B). During REMS, we found no differences between all four groups within the δ-band with the exception at 1 Hz. Here the frequency power of young MSA mice was significantly below values from old MSA and young control animals. For the in REMS dominantly present frequency band around 8–10 Hz (θ, α band), young MSA mice significantly showed increased power, compared to old MSA (hash marks) and young control animals (asterisks, Figure 5C). In contrast to WAKE active, the frequency power in the μ- and-β band of young MSA animals was only significantly increased to old MSA animals and not to both control groups.

### REM-A in MSA animals

**Figure 6:**
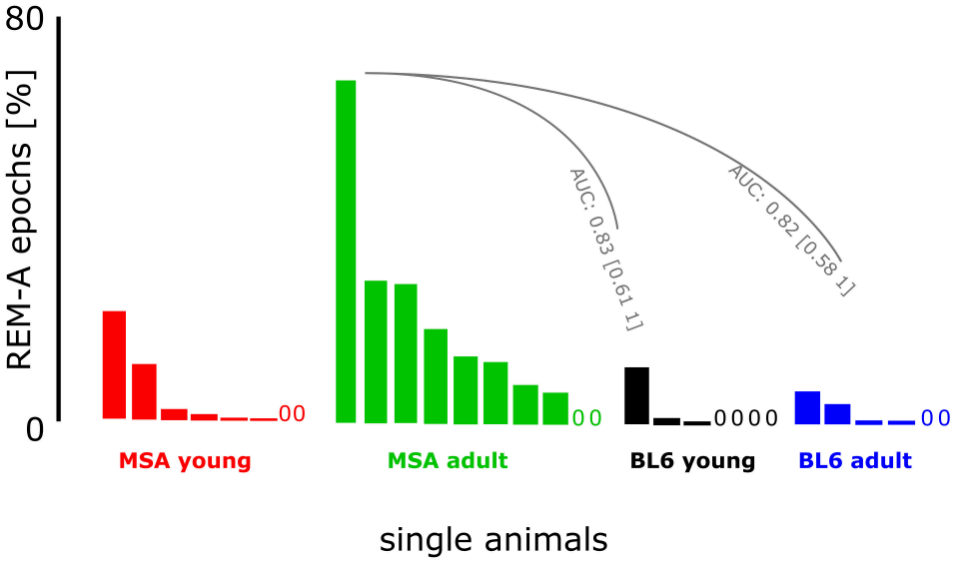
**Muscle activity during REMS in MSA mice and control animals**. The figure presents the proportion of REM-A. Each bar represents an individual animal within the experimental group. A “0” marks an individual animal without REM-A. We had to exclude one animal from the BL\6_10week_ group because of artifacts distorting the EEG. The animals in the control group expressed little REM-A in contrast to the MSA group. Especially the adult MSA animals showed high proportions of REM-A episodes. AUC analysis revealed strong and significant differences between the animals from the MSA_adult_ group and both BL\6 groups.

We detected small amounts of REM-A in 3 out of 7 young BL\6 and 4 out of 6 aged BL\6 mice. The maximum proportion of REM-A episodes was 12% in the young and 7% in the aged BL\6 group (percentage of REM-A in the total amount of REMS). In the MSA group, 6 out of 8 young and 8 out of 10 aged mice expressed REM-A with maximum proportions of 22% (young) and 69% (aged). The AUC analysis revealed that REM-A is strongly and significantly increased in the aged MSA animals when compared to young (AUC: 0.83; 95% Ci: 0.61–1) and old (AUC: 0.82; 95% Ci: 0.58–1) BL\6 mice. Figure 6 shows the occurrence and proportion of REM-A for each individual animal of all four experimental groups. Please refer to Supplementary Figure 3 for raw EEG- and EMG-recordings showing REM-A.

## Discussion

The development of MSA is commonly accompanied by sleep impairments such as disturbed sleep architecture, sleep fragmentation, RBD, and EDS, but there has been no sleep-related investigation of this neurodegenerative disorder in animal models of MSA, so far. Here, we describe the presence of several sleep impairments in the PLP α-SYN model for the first time. Furthermore, the expression of sleep impairments clearly correlated with age in mice, which strongly consolidates *Face Validity* in the PLP α-SYN model. Hence, we recommend to use the age-related sleep impairments as potential biomarkers to establish *Predictive Validity* with the present mouse model. We believe that our findings can contribute to future substance and drug screenings for MSA treatment.

### Sleep architecture

The general sleep/wake behavior of our young control animals (Figures 1, 2) is in accordance with previous findings in the literature on WAKE, NREMS, and REMS distribution in C57BL/6J mice (Tobler et al., 1997) and in C57BL/6N mice (Polta et al., 2013). Our old control mice expressed increased amounts of NREMS at the beginning of the active period, when compared to the young animals of the same group. Previous work on sleep patterns in adolescent mice revealed increased amounts of NREMS [postnatal P60-119, (Nelson et al., 2013)], while total sleep remained constant. Nelson and co-workers further described decreased REMS in early adolescence. We could not find such changes in REMS in our control animals, probably due to the age difference between the animals Nelson and co-workers used and the animals from our study. Additionally, the animals used by our colleagues were YFP-H mice, that have a (C57BL/6J × CBA) F1 background (Jackson, 2016). No comparative sleep analyses have been performed between these two lines so far.

To our knowledge only very few studies used aged mice in EEG studies so far. Silvani and co-workers for example recorded from hypocretin-deficient narcoleptic mice aged 10–11 month and found several changes in the sleep architecture (Silvani et al., 2014) between transgenic and control mice. But the study did not perform longitudinal EEG-recordings as performed in the present experiments.

Our control animals fit into the well-established picture of general sleep/wake behavior of mice. A comparison of the control animals with young and old MSA mice revealed no fundamental differences in WAKE and NREMS (intergroup comparison, Figure 1). As the genetic background of the PLP α-SYN model is the C57BL/6 breeding line (Kahle et al., 2002) we did not expect differences but performed these comparisons to ensure validity of our results.

The amount of REMS in old MSA mice was comparable to REMS levels of young and old control animals. This is in agreement with clinical findings on normal amounts of REMS in MSA patients (Vetrugno et al., 2004; Stanzani-Maserati et al., 2014). But old MSA animals spent significantly less amount in REMS than the young MSA mice. These differences in NREMS may be attributed to the differentiated expression of the disease with age. The significantly increased amounts of REMS in the young MSA animals have the potential to function as an early biomarker before other symptoms develop, at least in the PLP α-SYN mouse model, although further investigations are needed to reveal its strength. At present the use of this biomarker as a prodromal marker to the clinical situation is tempting, but is not applicable before the occurrence of early risk-factors such as RBD in patients. Around 50% of patients with idiopathic RBD are going to develop a synucleinopathy such as Parkinson's disease (PD), dementia, or MSA (Postuma et al., 2013).

The addition of observed elevated levels of REMS to the list of biomarkers at early stages of RBD-diagnosis may bear the potential to improve early diagnosis and to differentiate between developing synucleinopathies. Therefore, we suggest to add increased REMS to the list of other markers awaiting confirmation, as reviewed by Postuma et al. (2013). Elevated REMS in young MSA animals may as well serve as *Predictive Validity* for future drug screenings, as potential substances for treatment could reduce REMS to levels of control animals (and patients).

When compared with obstructive sleep apnea (OSA) patients, the sleep architecture of MSA patients is characterized by increased NREMS stages 1 and 2 and decreased NREMS stages 3 and 4 (Vetrugno et al., 2004) (*Note: In present clinical sleep research, the NREMS stages 3 and 4 are now summarized in NREMS stage 3*, Iber et al., 2007). In humans, NREMS stage 2 is mainly characterized by sleep spindles, while NREMS stage 3 is defined by the presence of dominant slow wave activity (Iber et al., 2007) (see also discussion below). In mice, sleep spindles *per se* are well defined (Vyazovskiy et al., 2004; Kim et al., 2012) but to date this criterion is not established yet to differentiate between individual sleep stages of NREMS in mice. So the differences in NREMS stage 1, stage 2 and stage 3 (stage 4) of MSA patients should not be used as parameters to establish sleep-related *Face Validity* in the PLP α-SYN model.

REMS latency seems to be decreased in MSA patients (Manni et al., 1993; Plazzi et al., 1997; Stanzani-Maserati et al., 2014). We also found decreased REMS latency in our MSA animals, compared to both control groups. We are aware that mice are nocturnal, polyphasic sleepers, facts hampering the transfer of clinical findings on shorter REMS latency to our results. So rather than supporting *Face Validity* of the PLP α-SYN mouse model for REMS latency, shorter REMS latency during the active period in mice may correlate to EDS sleepiness in humans diagnosed for MSA or PD (Ghorayeb et al., 2005; Moreno-López et al., 2011). This hypothesis is additionally supported by the fact that old MSA mice had significantly shorter WAKE bout lengths (coherent period of WAKE) in the active period (Figure 3B).

Data derived from a comparison of all possible transitions between the different vigilance states remained elusive, when all four groups of animals were compared (Figure 4). During the inactive period, only the young MSA animals were significantly different to the old control groups. At the moment we neither can connect this to distinct animal sleep/wake behavior, nor did we find an explanation in the clinical literature on MSA.

### Spectral analysis

We did not find different EEG PSD properties in the entire spectrum between young and old control animals for any vigilance state. During active WAKE (Figure 5A), the PSD of young MSA mice was higher at most frequencies compared to the old MSA mice and both control groups. Our results obtained from young MSA mice (Figures 5A,C) agree with findings from clinical studies. Patients diagnosed with iRBD expressed similar patterns of increased spectral power in the δ- and θ-band during WAKE and REMS (Fantini et al., 2003; Massicotte-Marquez et al., 2008; Iranzo et al., 2010; Brayet et al., 2015). In these clinical studies this spectral shift was a feature particularly observed - in patients that developed neurodegenerative disorders later on. The study of Iranzo and co-workers describes increased power in all frequency bands during WAKE and REMS, with most prominent shifts in the θ-band of patients developing mild cognitive impairments (MCI) (Iranzo et al., 2010). But in order to compare EEG signals from humans and mice, there are three critical aspects to consider: (1) The cortical topography is largely different. Additionally, the strongest frequency shifts described in the clinical studies were observed from central and frontal regions. We recorded EEG signals only from one electrode placed above the medio-frontal region of the mouse cortex. (2) EEG signals from single channel recordings in humans and mice may not be comparable on a one to one basis, because the size of the neuronal network generating the signal may be different. (3) The frequency range definition for the classical frequency bands varies for mouse and human EEG. (*Note that even particular frequency bands named identically in human and murine EEGs contain partly different corner frequencies of the bandpass filter*). Despite these differences we believe that the frequency shifts during the development of MSA-like symptoms strengthen *Face Validity* in the PLP α-SYN mouse model due to its similarity to clinical findings. Furthermore, we believe that these frequency shifts, as well as the mentioned increased amounts of REMS bear the potential to establish *Predictive Validity* with a mouse model for MSA for the first time. In our model, we would expect a potential treatment initiated at an early stage of the diseases to cause a shift of spectral power and/or REMS to baseline values. This assumption may be valid in other animal models for various other neurodegenerative diseases as well. Human studies linked an increase of spectral power in the δ- and θ-band and a shift of the frequency spectrum not only to RBD (as a precursor of MSA), but also to PD, Lewy body dementia (LBD) (Massicotte-Marquez et al., 2008) and Alzheimer's disease (Brayet et al., 2015). The PLP α-SYN mouse model may further be of use to investigate future treatment approaches to reduce MSA symptoms. Old PLP α-SYN mice receiving the treatment should show changes in their PSD back to levels of young MSA mice.

Our analysis of the SWA power revealed higher SWA power in young MSA animals than in the old ones and both control groups during the inactive period, indicating higher sleep intensity in the young animals (Figure 5B, 3–5 Hz). As sleep is regulated as a function of preceding WAKE (Borbély et al., 1981; Tobler and Borbely, 1986; Trachsel et al., 1986), SWA in particular serves as an index of sleep intensity (Franken et al., 1991). High levels of SWA may mirror increased sleep pressure in young MSA mice. It could also represent higher physical activity during preceding WAKE in young MSA mice. Lower levels of SWA could indicate lower overall sleep quality in old MSA mice. The lower SWA power may be coherent with clinical findings, where Parkinsonian patients showed lower sleep efficiency with disease progression (Diederich et al., 2005).

### REMS with muscle activity

REM-A, a lack of muscle atonia during REMS is one of the cardinal symptoms of RBD (Olson et al., 2000; Boeve et al., 2007; Boeve, 2010; Mccarter et al., 2012). This phenomenon, reported for 80–90% of MSA patients (Ghorayeb et al., 2002, 2005; De Cock et al., 2011) was also present in 80% of the old MSA mice (Figure 6). We also observed first signs of REM-A in young MSA mice that were not as manifested as in the old MSA group. To our knowledge, only one article described REMS without atonia in mice so far (Brooks and Peever, 2011). The authors could show that transgenic mice with an impaired GABA_A_- and glycine-receptor function exhibit the full spectrum of RBD symptoms including muscle activity during REMS. While Brooks and Peever analyzed the EMG signal in arbitrary 5 s epochs after processing the raw signal (Brooks and Peever, 2011), we always linked our 4 s EMG epochs (RMS-filtered) to its corresponding 4 s EEG epoch during analysis. This EEG/EMG linkage is based on a series of algorithms which decide in a first step between WAKE and sleep *per se* due to EMG activity in each 4-s epoch (Fenzl et al., 2007; Kreuzer et al., 2013, 2015). In a second step each previously scored sleep-state epoch is analyzed in the spectral and temporal domains of the EEG trace. This ensures a precise discrimination between quiet WAKE and sleep. Finally, all sleep-state epochs are analyzed in the spectral and temporal domains to discriminate between NREMS and REMS in each 4 s epoch (Kreuzer et al., 2015). In the present study REM-A was evaluated by manually rescoring each individual epoch assigned to REMS and each individual epoch originally assigned to WAKE which was flanked by at least three consecutive REMS epochs without muscle activity. Interestingly, our non-arbitrary approach and the approach applied by other authors (Brooks and Peever, 2011) closely resemble the clinical situation of MSA and/or RBD, supporting the appearance of REM-A in two different animal models.

Our procedure of scoring REM-A as described in the methods section may bear a subjectivity bias that we tried to overcome by-blinded scoring from two scorers. The use of neck muscle activity only to detect REM-A may hold some limitation when comparing potential REM-A in mice with clinical findings on REM-A during RBD, which includes more widespread body movements. Hence, further studies including a precise video analysis of mice during REMS are necessary. We further found a low proportion of REM-A in some of the control animals from both age groups. This could be a phenomenon which is also present in C57BL/6N mice and was so far excluded due to semi-automated scoring methods that automatically forbid muscle activity during REMS by default. Hence, REM-A episodes would have been missed by excluding this erroneous WAKE through manual re-scoring. This REM-A episodes in the controls may also be due to the mentioned subjective bias. But then, the subjectivity in blind re-scoring would account for all four experimental groups. So we are confident that occurrence of REM-A in mice can be established to strengthen *Face Validity* and *Predictive Validity* in the PLP α-SYN mouse model.

### Concluding remarks

The general sleep analysis of the PLP α-SYN mouse model for MSA revealed sleep impairments that have been described clinically. The change of spectral properties observed in the mouse model for MSA also agreed with results of studies conducted in humans. We further observed the phenomenon of REM-A, one of the key symptoms of RBD, in the animal model. Most important, the change in spectral properties as well as in REM-A probability was age dependent in the PLP α-SYN mouse model. Our data strengthen *face validity* of the PLP α-SYN model. The next step should be a critical evaluation for potential *Predictive Validity* in future studies.

Currently, research on MSA lacks potential compounds attenuating the course of or even curing the disease. Our findings may present an important step toward a valid animal model for future drug development. The described age dependency of REMS, the changes in the PSD, and the occurrence of REM-A episodes may have strong potential to serve as biomarkers for drug effects.

## Author contributions

LH, Performed parts of the experiments, wrote parts of the paper. TK, Performed parts of the experiments. MK: Developed analsysis software. EF, Performed parts of the experiments. GW: Wrote parts of the paper. NS: Wrote parts of the paper, designed parts of the study. TF: Designed the study, wrote parts of the paper, performed parts of the experiments

## Funding

This study was supported by the Austrian Science Fund (FWF) grants F4404 and F4414 and the Tiroler Wissenschaftsfonds (UNI-0404/1609).

### Conflict of interest statement

The authors declare that the research was conducted in the absence of any commercial or financial relationships that could be construed as a potential conflict of interest.
