# Supplementary material for: Distinct Parameters in the EEG of the PLP α-SYN Mouse Model for Multiple System Atrophy Reinforce Face Validity
Source: Front Behav Neurosci. 2017 Jan 10;10:252. doi: 10.3389/fnbeh.2016.00252 (PMC5222844; doi:10.3389/fnbeh.2016.00252)
Supplement: Supplementary file 1 [file Image1.PDF]

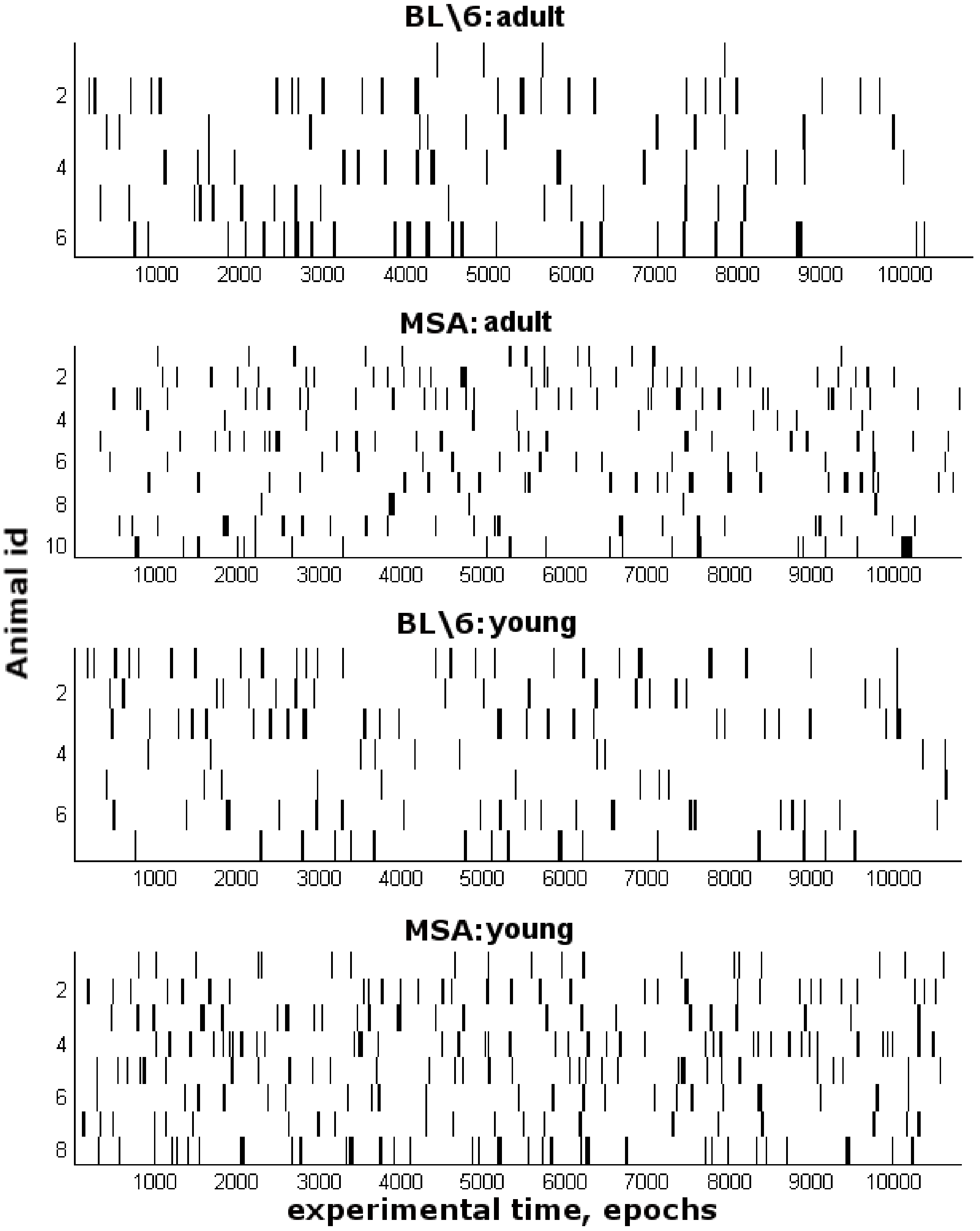

Supplementary Figure 1: Hypnograms from all four experimental groups showing REMS from the inactive period (lights on; X-axis: experimental time from hour 0 to hour 12, 10800 epochs). Each number within the Y-axis represents an individual animal from the corresponding experimental group. The black bars represent REMS episodes for each individual animal along the 12 hour period. The thicknesses of the bars correlate with the different durations of each individual REMS episode. The distribution of REMS episodes seems to be randomly distributed across the timeline of the recordings.
