# Supplementary material for: Distinct Parameters in the EEG of the PLP α-SYN Mouse Model for Multiple System Atrophy Reinforce Face Validity
Source: Front Behav Neurosci. 2017 Jan 10;10:252. doi: 10.3389/fnbeh.2016.00252 (PMC5222844; doi:10.3389/fnbeh.2016.00252)
Supplement: Supplementary file 2 [file Image2.PDF]

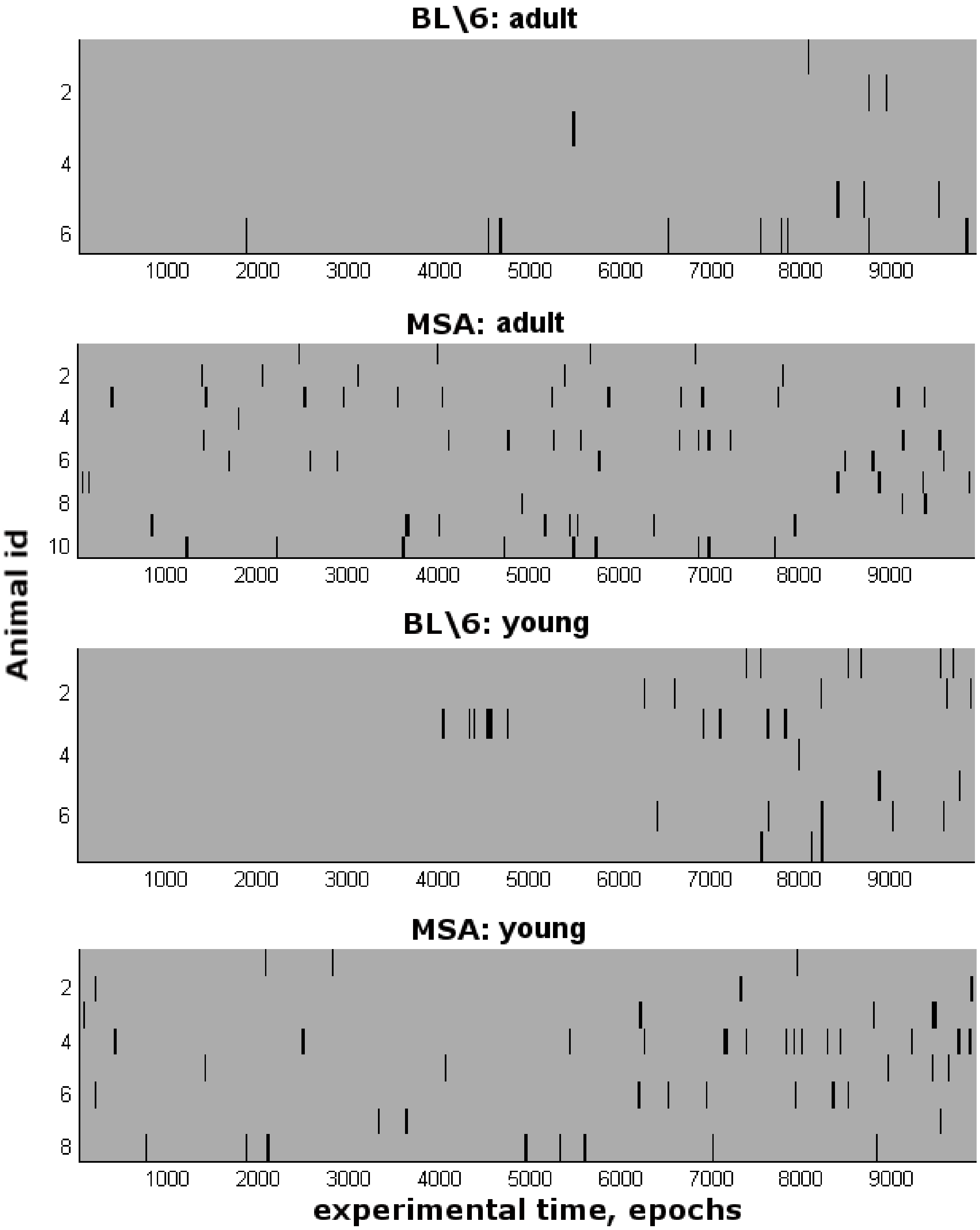

Supplementary Figure 2: Hypnograms from all four experimental groups showing REMS from the active period (lights off; X-axis: experimental time from hour 12 to hour 23, 9900 epochs). Each number within the Y-axis represents an individual animal from the corresponding experimental group. The black bars represent REMS episodes for each individual animal along the 12 hour period. The thickness of the bars correlates with the different durations of each individual REMS episode. Both control groups express REMS not before around 2000 epochs into the active period (ca. 130min after the transition from light to dark) while MSA groups show REMS directly after the transition from light to dark.
