# Supplementary material for: Distinct Parameters in the EEG of the PLP α-SYN Mouse Model for Multiple System Atrophy Reinforce Face Validity
Source: Front Behav Neurosci. 2017 Jan 10;10:252. doi: 10.3389/fnbeh.2016.00252 (PMC5222844; doi:10.3389/fnbeh.2016.00252)
Supplement: Supplementary file 3 [file Image3.PDF]

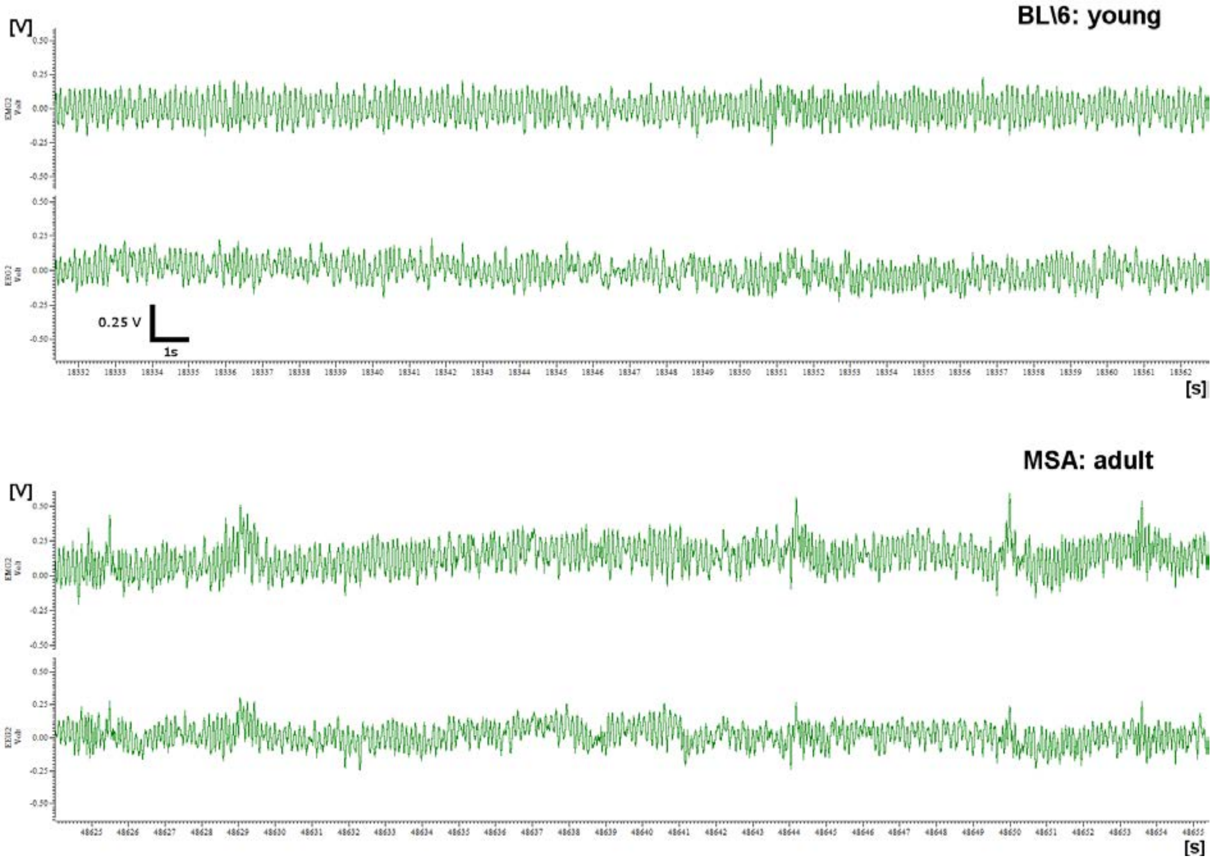

Supplementary Figure 3: EEG and EMG examples during REMS showing REM-A events in an adult MSA mouse (bottom graph). The top trace in each graph represents EMG recordings and the bottom trace in each graph represents the corresponding EEG recordings. Clearly in the representative example from the control group the EEG has a very typical waveform and amplitude for REMS (mouse). The EMG of the representative control mouse has no signs of movements or muscle twitches. In contrast the representative EMG trace from the MSA mouse clearly shows several signs of body movements /muscle activities during the otherwise very typical REMS EEG/EMG recordings. All traces represent raw recordings (amplification: 1000x; bandpass filter: 0.5-100Hz); X-axis: experimental time in seconds (30 second samples are plotted); Y-axis: waveform amplitude in Volts.
